# An Innovative Approach to Occupational Risk Assessment in OHS: A Case Study on the Verification of the ALrisk Model in Manufacturing Enterprises in Slovakia

**DOI:** 10.3390/ijerph22050757

**Published:** 2025-05-12

**Authors:** Alena Kuricová, Mária Hudáková, Samuel Kočkár, Katarína Hollá

**Affiliations:** Faculty of Security Engineering, University of Žilina, 010 26 Žilina, Slovakia; maria.hudakova@uniza.sk (M.H.); kockar@uniza.sk (S.K.); katarina.holla@uniza.sk (K.H.)

**Keywords:** occupational risk assessment, workplace safety, ALrisk model, manufacturing enterprises, occupational health and safety, risk management

## Abstract

The issue of occupational health and safety (OHS) is currently a pressing and essential challenge for improving production processes and workplace environments, particularly in manufacturing enterprises. With increasing demands for efficiency and workplace safety, it is crucial to implement innovative approaches that enhance accident prevention and safeguard employees’ health. These approaches contribute to the long-term sustainability of enterprises and reduce costs associated with workplace injuries and occupational diseases. The core focus of this article is to present the ALrisk model for OHS risk assessment and management, outlining its key components, as well as the results and benefits of its verification in specific job positions within manufacturing enterprises in Slovakia. The study employed scientific methods, along with risk identification, analysis, and workplace condition assessment methods, in the development and verification of the ALrisk model. These methods contributed to a more precise identification of factors endangering employees’ safety and health and enabled the formulation of solutions for their mitigation. The application results indicate that the proposed model provides a more effective method for assessing occupational risks, thereby enhancing prevention—reducing health hazards for employees and improving overall workplace safety. The article offers practical insights into the application of the ALrisk model as an innovative and systematic approach within the specific conditions of manufacturing enterprises. The findings of the study serve as a valuable resource for OHS managers and senior employees seeking to improve workplace safety and accident prevention within their production processes. Moreover, the results are beneficial for other professionals engaged in OHS, particularly in the assessment and management of occupational risks, not only in Slovakia but also across European countries.

## 1. Introduction

Given the constantly evolving work environment, technological advancements, and increasingly stringent legislative requirements, it is essential to assess and minimise risks that could lead to workplace injuries, occupational diseases, or material losses [1]. Additionally, in the context of competition, effective risk management in OHS is playing an increasingly significant role from the perspective of strategic business management [2]. Systematic risk assessments are crucial for identifying hazards and developing appropriate prevention strategies to mitigate physical, material, and environmental damage [3]. The COVID-19 pandemic has further heightened the urgency for businesses to implement effective OHS measures, as it directly impacts workforce productivity and morale [4]. Regular risk assessments help identify potential hazards, thereby reducing the likelihood of workplace injuries and illnesses [5]. Companies operating on an international scale face diverse regulatory frameworks, making timely risk assessments essential for ensuring consistent compliance [6,7]. According to multiple authors, such as [8,9,10,11], organisations that systematically implement risk elimination measures not only achieve a higher level of compliance with legislative requirements but also experience long-term cost reductions related to workplace accidents and enhanced market competitiveness. Effective OHS risk management can increase productivity and reduce downtime caused by workplace incidents [12]. Moreover, socially responsible enterprises that actively engage in ensuring workplace safety gain an advantage in the form of a positive corporate image and increased trust from business partners, investors, and the public [13]. Occupational risk assessment represents a key component of corporate management, directly influencing employee protection, production process continuity, and overall organisational sustainability. The integration of modern approaches and analytical tools provides businesses with a systematic framework for identifying threats, assessing their severity, and implementing effective preventive measures [7,14,15]. Emphasising proactive risk management fosters the development of a safety-oriented workplace culture, where employees are motivated to adhere to safety standards, ultimately reducing the likelihood of unforeseen incidents and their negative impacts on the organisation.

Given these considerations, it is essential for businesses to devote increased attention to occupational risk assessment in OHS and continuously innovate their approaches to hazard prevention and management. The long-term goal of effective workplace risk assessment is not only to protect employees’ health and lives but also to enhance overall business performance and ensure the sustainability of operations in a competitive environment.

After comparison with other approaches that are implemented in practice, we identified the need to create a step-by-step procedure that is systematic, descriptive, and illustrates all the connections between individual steps.

By consistently applying the ALrisk model according to the defined steps, all risks are assessed, which contributes to a reduced number of occupational accidents, hazardous situations, and minor injuries. The ALrisk model places emphasis on involving employees in the entire risk assessment process, which is not a given everywhere. This particularly concerns employee representatives who participate in the process of assessing and verifying the outputs of risk assessments and the measures that are established for unacceptable risks.

The ALrisk model represents a systematic and practically verified approach to the assessment and management of risks in the field of occupational health and safety (OHS), which enables companies to identify specific risks at individual job positions, quantify their level, and propose effective preventive measures. In practice, this means a reduction in the occurrence of occupational injuries and diseases, which is directly reflected in improved employee health, lower absenteeism, and higher productivity. At the same time, the model allows companies to optimise OHS processes, leading to cost savings related to accidents, penalties, and production losses. Verification of the model in specific manufacturing companies has shown that its application led to measurable improvements not only in employee health but also in the efficiency of workplace management, which contributes to the long-term sustainability and competitiveness of the company.

Within this article, the research focuses primarily on the first key part of the ALrisk model—risk assessment—which represents the analytical basis for effective handling and management of OHS risks. Although the ALrisk model in its complete form also integrates the subsequent steps of risk management, the subject of this study is primarily the process of identification, analysis, and evaluation of risks at specific job positions.

The application of the ALrisk model in several manufacturing companies and at various job positions confirms its practical usability and flexibility. For employees, it represents a significant component in OHS documentation, as it allows for better orientation in the procedure of “how to carry out risk assessment in the workplace”.

The results presented in this article not only provide convincing evidence of the practical effectiveness of the ALrisk model but also offer a comprehensive, methodologically grounded, and scientifically verified approach to addressing the issue of occupational risks in the context of OHS in Slovakia and abroad.

The article is original primarily in the development and verification of the ALrisk model for specific job positions in OHS within manufacturing enterprises. Its unique approach lies in the innovative and systematic assessment of occupational risks for the specific professions of ‘machine operator’ and ‘Operator of heat exchanger and homogenisation,’ thereby expanding the practical applicability of the ALrisk model in industrial environments. The article provides a detailed methodological framework that enables a more effective evaluation and mitigation of occupational risks, significantly contributing to the reduction of workplace injuries and occupational diseases. Furthermore, it presents a practical validation of the model under real-world conditions in manufacturing enterprises in Slovakia, assessing its impact on reducing costs associated with workplace accident prevention, occupational disease management, and improving the efficiency of work processes. This research not only offers new insights into occupational risk assessment but also provides concrete recommendations for businesses considering the implementation of the ALrisk model in other segments of industrial production.

### Terminology

Occupational risk assessment is an integral part of any enterprise that prioritises the safety and well-being of its employees. According to OSH WIKI (2022) [16], risk assessment can be defined as the process of evaluating health and safety risks for workers arising from workplace hazards. Bejinariu et al. (2024) [5] describe risk assessment as a systematic process involving the identification, analysis, and evaluation of risks and hazards. Rantala et al. (2022) [9] state that risk assessment is an effective procedure aimed at reducing potential workplace risks. This means that a competent managerial employee is responsible for conducting risk assessments and proposing measures to eliminate and control workplace risks in any given situation [8]. Hollá et al. (2017) [17] assert that the fundamental principle of risk assessment is the identification of all potential hazards in the workplace and their effective management to minimise possible harm to employees’ health and its related consequences. According to EU OSHA (2007) [18], the primary objective of workplace risk assessment is to protect employees’ health and safety. Risk assessment helps to minimise harm to workers and the environment caused by workplace activities. Kimmel and Vu (2001) [19] highlight that risk assessment also supports maintaining business competitiveness and operational efficiency. Moreover, it raises awareness of existing hazards, ensuring that employees are informed about workplace risks based on the findings of the assessment. The National Labour Inspectorate of Slovakia (2025) [20] explains that before conducting a risk assessment, it is essential to gather information about the enterprise to better understand the activities employees perform and the hazards they encounter, such as handling heavy loads, exposure to chemicals, ergonomically unsuitable workplaces, or working with designated technical equipment (DTE). Additionally, it is important to consider the work equipment used, working conditions, and potential exposure to specific risks. Other key factors include the number of employees, the size of the enterprise, and its sector—whether it is involved in manufacturing or service provision. Adem (2022) [8] supports this conclusion, asserting that the information gathered about an enterprise and its employees is crucial for properly defining risk assessment criteria within the organisation.

The assessment and subsequent management of occupational risks are mandated by legal regulations in every country. For an organisation to function properly, risk assessment must be integrated into its documentation and overall business philosophy. For example, in the Slovak Republic, occupational risk assessment is a legal requirement under Act No. 124/2006 Coll. on Occupational Health and Safety (hereinafter referred to as the OHS Act) [21] (Act No. 124/2006 Coll.). This law stipulates that employers must ensure workplace health and safety by implementing necessary measures: evaluating risks within the organisation, providing OHS training for employees, documenting records of workplace injuries, involving employees and their representatives in discussions on OHS, and other measures as required by national legislation in each country. These obligations also apply directly to employees, requiring them to properly use equipment and remain informed about hazards and deficiencies in safety measures. The law further emphasises that OHS is an essential part of corporate management and should be integrated into a company’s systematic management framework (Occupational Health and Safety Concerns Us All, 2025) [22]. From this, it follows that risk assessment is not only a legal obligation but should also be an integral part of an OHS management system. Enterprises must primarily comply with legally established OHS requirements while also implementing managerial systems to ensure more effective compliance with all safety regulations and the achievement of business objectives. According to Ramos et al. (2015) [23] and Podgorski (2010) [24], an OHS management system represents a component of a company’s overall management system that establishes an OHS framework, manages health and safety risks, and, through its implementation, achieves effective business outcomes and objectives in alignment with OHS management requirements.

Occupational risk assessment in OHS is a critical process that varies across industries and methodologies. Established procedures typically incorporate a combination of quantitative and qualitative approaches tailored to specific workplace conditions. The following sections outline key methodologies and their applications in occupational risk assessment: Komzolov et al. (2024) [25] emphasise that there is no universal approach to occupational risk assessment in OHS. Employers select methodologies based on industrial processes and existing health management systems, leading to diverse practices without standardised risk assessment procedures. They propose a method that includes calculating costs associated with ensuring employee health and safety, aiding in justifying risk response activities. Kvitkina et al. (2023) [26] developed a risk assessment method that involves compiling a list of risk factors, determining probability and damage coefficients, calculating occupational risk levels, classifying assessment results, and formulating workplace risk mitigation measures. This approach utilises mandatory reporting documents to reflect actual working conditions, enhancing the relevance of the assessment. Gerasimenko (2023) [27] recommends conducting occupational risk assessments using the matrix method and the Fine–Kinney method, which facilitate hazard evaluation based on probability and exposure. These methods incorporate linear and quadratic interpolations for more precise risk level assessments. Bejinariu et al. (2024) [5] outlined standardised occupational risk assessment procedures in OHS, suggesting that an effective process should include a company and work system description, the application of an assessment method using Microsoft Excel, and the presentation of OHS measures to implement a prevention and protection plan. Golinko et al. (2022) [28] introduced a semi-quantitative occupational risk assessment method, incorporating factors such as accident probability, impact duration, accident severity, personnel competency, and management effectiveness. Their framework includes criteria for hazard identification and recommendations for improving safety based on audit findings. Similarly, Petchenko (2023) [29] highlights that implementing workplace safety audits in Ukrainian enterprises is crucial for identifying and addressing safety issues, thereby improving working conditions and ensuring employee safety through continuous enhancement of the occupational safety management system. According to Rantala et al. (2022) [9], Finland’s prevailing practice involves the use of the Workplace Risk Assessment Workbook, which includes checklists for hazard identification and a risk matrix-based decision protocol. These methods form part of standardised occupational risk assessment procedures in OHS. Khamidullina and Nayanov (2022) [30] highlight the bow-tie method as one of the 13 recommended approaches for occupational risk assessment, focusing on high-risk operations, safety barrier analysis, and statistical data utilisation to identify common causes of accidents, particularly in oil extraction. Adem (2022) [8] proposed a risk analysis techniques assessment manual for OHS, prioritising selection criteria using the Analytic Hierarchy Process (AHP) for effective decision-making in risk assessment. Kuzheleva et al. (2024) [11] developed a methodology to enhance the efficiency of corporate occupational safety management systems, focusing on risk assessment and management to improve working conditions and reduce exposure to environmental hazards. Key steps include creating a unified risk factor register, determining numerical risk values for individual workplaces, and identifying the most significant risk factor groups. For high-risk workplaces, measures are proposed to reduce environmental factor impacts on employees, contributing to the overall improvement of OHS management systems. Nitescu et al. (2023) [15] introduced the MAXM method for OHS risk assessment, evaluating hazards to protect workers from workplace threats, determining the severity and probability of hazardous outcomes, and considering both acute and chronic effects on worker health and safety. Nunes (2010) [31] focused on the use of fuzzy logic in occupational risk analysis, overcoming the limitations of traditional methods in handling uncertainty and subjective factors. He proposed a method that assesses risks based on occurrence probability and consequence severity, where the fuzzy system allows for more accurate interpretations using linguistic variables (e.g., ‘low’, ‘medium’, and ‘high’). This approach represents an innovative application of fuzzy logic in occupational risk analysis, enabling more precise and efficient identification of risk situations and the implementation of effective preventive measures.

The fact that there are no fixed rules on how OHS risk assessment should be conducted is also supported by experts from the EU Commission for OHS [32]. There are numerous methodologies, methods, and procedures for risk assessment, and different approaches may be suitable under various circumstances. However, two key principles should always be considered in risk assessment: ensuring that all relevant hazards and risks are addressed and, after identifying a risk, beginning the evaluation with the fundamental question of whether the risk can be eliminated. According to the European guide Fundamentals of Risk Assessment [18], the risk assessment process can be carried out as follows: collecting information, identifying hazards and threats, assessing risks arising from threats (estimating the probability and severity of consequences and determining whether the risk is acceptable), planning measures to eliminate or mitigate risks and repeating the risk assessment, and documenting the assessment. OIRA EU-OSHA (2025) [32] states that for most businesses, particularly small and medium-sized enterprises (SMEs), a step-by-step approach based on risk management principles has proven effective: Hazard and threat identification—recognising elements in the workplace that could cause harm and identifying workers exposed to these hazards. Risk assessment and prioritisation—estimating existing risks (severity and probability of potential harm) and ranking them by importance. Decision-making on preventive measures and implementation—identifying appropriate measures to eliminate or control risks and integrating these measures into workplace safety plans. Implementing preventive and protective measures through a structured priority-setting plan. Monitoring and review—reassessing risk evaluations at regular intervals to ensure they remain up to date. The EU Commission has also developed the Risk Assessment Methodology for Occupational Health and Safety to provide practical support for implementing risk assessment requirements from the Framework Directive [16,33]. For specific risk assessment steps, the general framework provided by ISO 31001:2018 is recommended [34]. This standard describes risk assessment as a structured process consisting of the following: Risk identification—the process of identifying, recognising, and describing risks. Risk analysis—the process of understanding the nature of risk and determining its level. Risk evaluation—comparing the results of the risk analysis against risk criteria to determine whether the risk and/or its magnitude are acceptable or tolerable.

One of the ways to manage risks within an occupational health and safety management system (OHSMS) is by following the approach outlined in ISO 45001:2018 (Occupational Health and Safety Management Systems—Requirements with Guidance for Use) [35]. The primary purpose of risk management in OHS, as defined by this standard, is to prevent workplace injuries and occupational diseases while improving employee safety and health. The fundamental principle of ISO 45001:2018 is the identification of all potential risks in the workplace and their effective management to minimise possible harm to employees and its associated consequences [35]. According to Karanikas et al. (2022) [36] risk management is at the core of ISO 45001:2018, as it helps organisations not only comply with legal requirements but, more importantly, protect the health and safety of employees. ISO 45001:2018 provides a systematic approach to identifying, assessing, and managing occupational health and safety (OHS) risks, thereby enabling organisations to create safer working environments. The standard recommends the following steps in risk management: identifying hazards, assessing OHS risks and additional OHSMS-related risks, complying with legal requirements related to OHSMS, and planning appropriate measures. For specific risk assessments, ISO 45001:2018 recommends alignment with ISO 31000:2018 [34,35]. This approach is also supported by [23], which, based on an analysis of the opinions of company technicians, highlighted key aspects of integrating risk management into corporate OHSMS while considering both technical and human factors.

For an effective assessment of occupational risks in OHS, various methods, techniques, and tools are recommended. According to [8,17,30,37], the most commonly used tools include checklists, risk matrices, tree diagrams (e.g., Ishikawa diagrams, fault tree analysis (FTA), event tree analysis (ETA)), bow-tie analysis, HAZOP (hazard and operability study), and FMEA (failure mode and effects analysis). Specialised software tools also play a crucial role by automating the risk assessment process. These tools provide comprehensive functionalities, such as risk database management, report generation, and integration with other systems, enhancing the efficiency and accuracy of risk evaluation.

According to several authors, such as Felknor et al. (2021) [38] and Klimová et al. (2017) [39] continuously improving OHS standards through risk management leads to reduced losses, higher productivity, increased efficiency, and improved work quality. These factors positively impact overall business performance and, most importantly, employee safety. Rudakov et al. (2021) [40] and Pinto et al. (2012) [41] state that the primary responsibility of all enterprises—whether small, medium, or large—is to enhance occupational health and safety conditions, with an emphasis on prevention and the application of risk management practices. According to Matkovčíková (2017) [42] properly designed and implemented workplace risk assessment can protect employees and reduce risks associated with their work. Similarly, Bibire et al. (2020) [10] highlight the importance of risk management as a key factor in preventing adverse events in OHS. Effective risk management contributes to a lower incidence of workplace injuries, improved workplace safety and health, enhanced productivity, and increased business competitiveness.

Based on the aforementioned baseline analysis, it can be concluded that despite the existence of various established methodologies for occupational risk assessment in OHS, which provide structured approaches, challenges in standardisation and implementation across different industries persist. This underscores the need for continuous research and development in the assessment and management of occupational risks.

## 2. Methodology

In the years 2021–2023, research was carried out within the project supported by the Slovak Research and Development Agency (APVV), APVV-20-0603: “Development of risk assessment tools for selected enterprises and professions in the Slovak Republic in accordance with EU requirements”. The main objective of the research was to create a tool for risk assessment of selected enterprises and professions in the Slovak Republic in accordance with EU requirements, in order to reduce occupational accidents.

The research consisted of the following parts:

1. Definition of the subject, aim of the research, and formulation of hypotheses,
2. Conducting an initial analysis of existing studies and theories,
3. Defining the research methodology,
4. Qualitative (interviews, discussions, consultations) and quantitative (questionnaire survey) data collection,
5. Processing the results and proposing a solution,
6. Verification of the proposed solutions,
7. Training of enterprises in the use of the proposed solution,
8. Implementation into the specific conditions of enterprises in Slovakia,
9. Summary and evaluation of the main benefits.

The aim of this article is to present the results of the proposed and verified ALrisk model for specific job positions in OHS within manufacturing enterprises in Slovakia. The proposed ALrisk model is based on an innovative and systematic assessment of occupational risks for the specific professions of ‘machine operator’ and heat exchanger and homogenisation unit operato. It evaluates the model’s impact on reducing costs associated with workplace accident prevention and improving the efficiency of work processes. The main purpose of the proposed model is to achieve a reduction in occupational accidents in manufacturing enterprises for the job positions of machine operator and heat exchanger and homogenisation unit operator.

To achieve the stated objective, the following methods were employed:

1. Analysis of statistical data—Statistical data from the National Labour Inspectorate and ESENER surveys served as a basis for the research, highlighting the necessity of an effective approach to OHS management and the assessment and management of occupational risks.
2. Analysis and comparison of existing methodologies—Existing occupational risk assessment methodologies were analysed and compared to develop the ALrisk model.
3. Survey analysis—The results of a conducted survey on occupational risk assessment, carried out within the framework of the scientific project APVV-20-0603, were examined.
4. Consultations with experts and academics—Discussions with OHS specialists at both the European and Slovak levels contributed to the development of the model, ensuring its systematic structure and logical sequencing of phases and steps.
5. Statistical tools and software support—Tools such as Excel and the web-based platform OiRA were utilised for designing and verifying the model.
6. Checklists, point-based methods, and risk catalogues—These were applied during the verification process in real-world conditions, enabling an objective assessment of occupational risks as an integral part of the ALrisk model’s risk evaluation framework.

The methodology of the article consists of the following steps:

1. Collection and analysis of statistical data—Gathering data from the National Labour Inspectorate and ESENER surveys. These data served as a foundation for the research, emphasising the need for effective occupational risk assessment and management in OHS.
2. Baseline analysis of existing methodologies—Reviewing various occupational risk assessment methodologies based on scientific articles, studies, and research. Comparing these methodologies to identify the most effective approaches.
3. Expert consultations, safety inspections, and discussions with practice during the model proposal—the proposal of the ALrisk model was consulted with several experts from both academic and corporate sectors to ensure its practical feasibility and professional accuracy. The research involved, for example, Bc. Miloš Eichler, Ing. Katarína Hannelová, Ing. Anna Cidlinová, PhD, Ing. Štefan Káloši, PhDr, David Michalík, PhD, DBA, Ing. Marek Bárdy, PhD, Ing. František Mrásko, and other experts active in the field of OHS. Representatives of the assessed enterprise also played an important role, with 10 expert meetings held at individual plants (e.g., Žilina, Bratislava, Košice, Martin, Trnava, Zvolen). These discussions contributed to the optimisation of the model based on real operational conditions and the needs of the enterprises.
4. Integration of selected methods into the proposed model—Utilising findings from OHS-related research. Incorporating information on risk assessment obtained within the framework of the APVV scientific project.
5. Systematisation and development of the ALrisk model—Consulting with experts and academics in the field of OHS. Ensuring a logical structure and a clear arrangement of the model’s phases.
6. Verification of the model using software tools—testing of the ALrisk model was carried out in real conditions of selected manufacturing companies in Slovakia. Managers, safety technicians and employees working in the given job positions were involved in the assessment. Tools such as Microsoft Excel were used to verify the functionality of the model, where a checklist and risk catalogue were created directly. The OiRA web platform served as an inspirational platform in the verification of the ALrisk model, focusing on micro and small enterprises. These tools proved suitable especially in terms of practical applicability and accessibility. Excel enabled clear processing and comparison of data between individual operations and job positions, while OiRA provided an interactive space and inspirational perspective. The verification results demonstrated a high degree of applicability and benefit of the proposed model in practice, thus confirming its effectiveness in risk reduction and improvement of occupational safety.
7. Evaluation of model verification in practice—Using checklists, point-based methods, and risk catalogues for assessing and objectively evaluating occupational risks.

## 3. Results

To achieve the objectives of this article, the following results were processed:

1. Development of the ALrisk model—A framework for occupational risk assessment.
2. Evaluation of key results from the verification of the proposed model for the job position of machine operator in a manufacturing enterprise.
3. Evaluation of key results from the verification of the proposed model for the job position operator of heat exchanger and homogenisation in a manufacturing enterprise.

The development of the ALrisk model and its graphical overview emphasised a clear and comprehensible presentation of the main component—risk assessment and its two phases. The results obtained from the application provide valuable insights, demonstrating that the application of the ALrisk model is effective and applicable to any manufacturing process. The successful implementation of the model’s approach indicates that timely risk assessment within enterprises can significantly reduce both the likelihood and impact of workplace accidents involving employees.

### 3.1. The ALrisk Model Proposal for Occupational Risk Assessment and Management in OHS

The ALrisk model for occupational risk assessment and management in OHS represents a structured process that provides a comprehensive approach to implementing OHS management in the assessment and control of risks. The responsible person within the enterprise, who oversees OHS management and, consequently, the assessment of occupational risks for all employee activities, can fulfil their obligations by following the prescribed steps within the phases of the ALrisk model. This ensures the proper execution of duties in accordance with the established procedure. By adopting this approach, one of the general employer obligations is fulfilled [21,43].

The core component of the ALrisk model—risk assessment—consists of two consecutive phases, each comprising specific steps. The first main part, Risk Assessment, includes the following phases: 1.1 Phase—Preparation of the OHS Risk Assessment Process 1.2 Phase—Implementation of the OHS Risk Assessment Process [43,44].

1.1 Phase—Preparation of the Risk Assessment Process includes the following steps:

1.1.1 Step—Definition of Criteria.

1.1.2 Step—Material and Personnel Provision.

1.1.3 Step—Familiarisation with the Job Position/Work Environment.

1.1.4 Step—Inspection of the Current Operational State.

1.1.5 Step—Evaluation of the Current Operational State. Process as per [43,44].

1.2 Phase—Implementation of the OHS Risk Assessment Process includes the following steps:

1.2.1 Step—Identification of Hazards and Threats.

1.2.2 Step—Analysis of Hazards and Threats.

1.2.3 Step—Risk Assessment.

1.2.4 Step—Determination of Measures.

**Figure 1:**
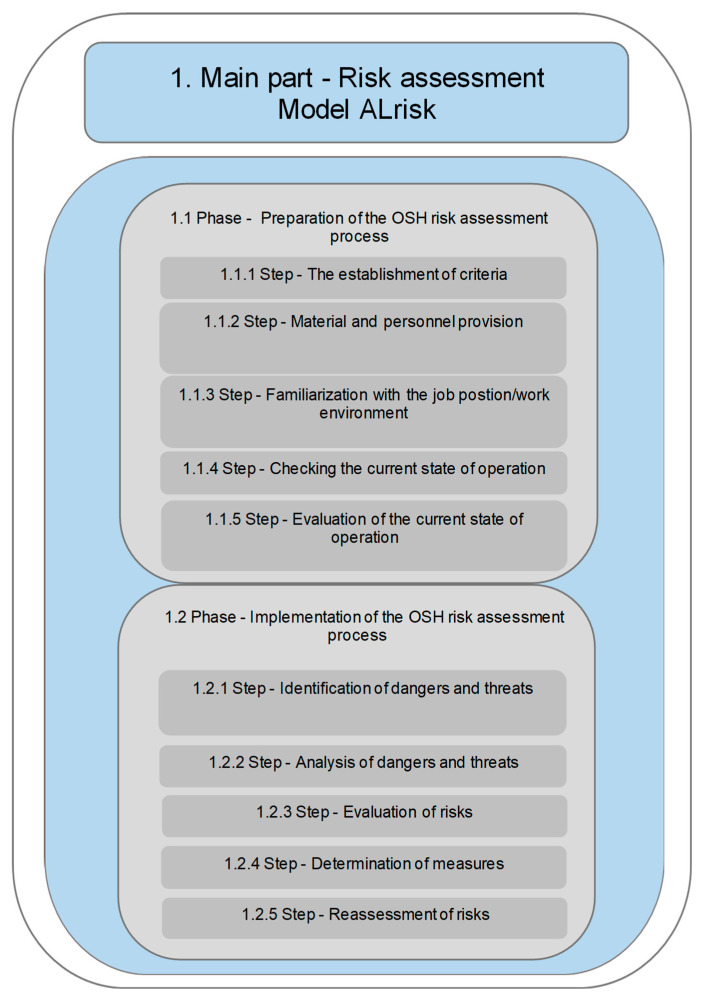
First main component of the ALrisk model—1. Main component: Risk Assessment [42].

1.2.5 Step—Reassessment of Risks. Process as per [43,44]. Figure 1 illustrates the structure of the ALRisk model [42].

Phase 1.1 represents the preparatory phase, which includes several steps that must be completed before the actual implementation of risk assessment. Among the essential steps, Step 1.1.3—Familiarisation with the Job Position/Work Environment and Step 1.1.4—Inspection of the Current Operational State are regarded as the two most crucial steps of Phase 1.1. This is because understanding the job position, its specific characteristics, and the specifics of the work environment, along with on-site inspections before the commencement of Phase 1.2, are key to properly setting the methods for conducting the OHS risk assessment and effectively achieving the established objectives and outcomes of the OHS risk management process for the selected job position. Phase 1.2 of the ALrisk model represents the phase that includes the direct steps involved in carrying out the OHS risk assessment. Each step within Phase 1.2 is essential and significant, as they are interdependent, and none can be omitted from the process. If these steps are correctly implemented within an enterprise according to the prescribed procedure, it is highly likely that the risk assessment process will be successfully integrated and will yield positive results in OHS management [43].

#### Originality and Significance of the Proposed ALrisk Model

From a theoretical perspective, the uniqueness of the ALrisk model is demonstrated through its integration of existing methodological approaches, such as the EU-OSHA risk assessment process and the KatAlSam method, which are already utilised in practice. The distinctiveness of the model is also recognised by various enterprises in its systematic approach, where the assessor follows the phases step by step. Additionally, the ALrisk model was designed based on valuable theoretical and practical insights from OHS professionals, further strengthening its applicability. The model’s originality is also grounded in its alignment with ISO 31000:2018—Risk Management Process [34]. Specifically, the section “Scope, Context, and Criteria” from ISO 31000:2018 directly corresponds to Phase 1.1—Preparation of the OHS Risk Assessment Process within the ALrisk model’s first main component—Risk Assessment [34]. Furthermore, the ISO 31000:2018 sections on risk treatment, risk recording and reporting, monitoring, and review are reflected in the second main component—Risk Management, particularly within Phase 1.2—Monitoring of OHS Risks [34]. In the ALrisk model, the risk review process has been separated and represents one of its supporting components. The communication and consultation aspect of ISO 31000:2018 is expanded within the ALrisk model by incorporating joint meetings and advisory sessions, forming its second supporting component [34]. From a practical perspective, the proposed model was verified to enhance OHS, with a particular focus on risk assessment and the prevention of workplace accidents, occupational diseases, and other work-related health impairments. The uniqueness of the verification process lies in its comprehensive workplace inspections, photographic documentation, preparation of procedures and methods for the application of risk assessment, and direct discussions with employees in the assessed job positions. Additionally, the ALrisk model was directly integrated with tools developed by the European Agency for Occupational Safety and Health (EU-OSHA) within the OiRA web platform, further reinforcing its practical applicability and effectiveness.

### 3.2. Evaluation of Key Results from the Verification of the Proposed Model for the Job Position of Machine Operator in a Manufacturing Enterprise

**Figure 2:**
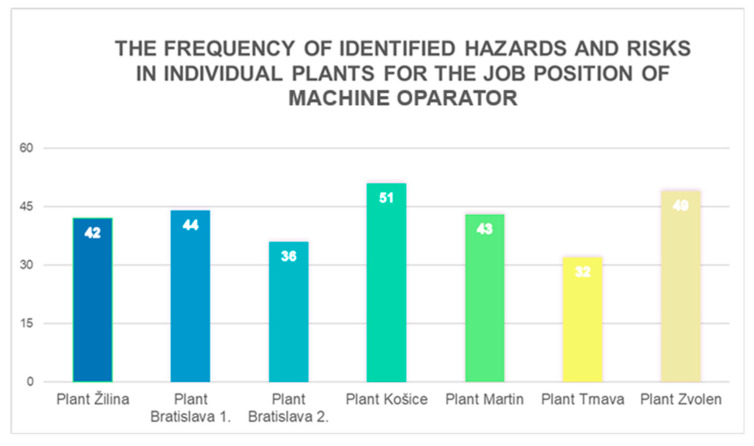
Frequency of identified hazards and risks in individual plants and operational sites of the manufacturing enterprise for the job position of machine operator (Authors).

The job position of a machine operator is present in every operational unit of the manufacturing enterprise, which comprises six plants and seven operational sites across Slovakia. The ALrisk model was applied in each of these operational sites. The role within the heating plant is crucial, requiring specialised knowledge and skills to operate technological equipment involved in energy production and conversion. To perform this role, employees must undergo proper training, possess a valid permit for the operation of designated technical equipment, and have the necessary professional knowledge and skills for this specific job position. The machine operator’s responsibilities include ensuring plant operation, performing maintenance, and adhering to organisational safety regulations, standard operating procedures, and emergency protocols. The employee primarily works in the engine room, boiler room, and technology control room, with occasional presence in the control centre. During work, the machine operator conducts regular site inspections, monitoring the technological infrastructure and checking equipment functionality. These rounds are performed at regular intervals or as needed in case of equipment failures. The operator directly handles technical issues within the facility. Machine operators are exposed to multiple occupational hazards, including noise, hot media, vibrations, cold stress, heat stress, working at heights, exposure to hazardous substances, unsafe surfaces, confined spaces, visual display unit strain, and musculoskeletal disorders (MSDs) due to repetitive tasks. They operate all types of designated technical equipment and use assigned and fully functional personal protective equipment (PPE). Operators typically work alone during shifts, though in cases of serious technical malfunctions, other colleagues, such as technology control operators and production supervisors, may be present at the workplace or in the control room. During the OHS risk assessment and management process, multiple internal enterprise members participated. The process involved the following roles: OHS Manager, OHS Specialist, Supervisor of Production Operations, Production Resources Manager, Machine Operator, and OHS Technician. Communication with employees at MH Teplárenský Holding, a.s. took place in person, via email, and by telephone throughout all stages and phases of the proposed ALrisk model. Across all six plants and seven operational sites, a total of 297 hazards and risks associated with the machine operator job position were identified in the work environment. The distribution of identified hazards across the different operational sites is illustrated in Figure 2.

In the Žilina plant, 42 hazards were identified. In Bratislava, 44 hazards were recorded at Plant 1, and 36 hazards at Plant 2. The Košice plant had 51 identified hazards, while the Martin plant recorded 43 hazards. In Trnava, 32 hazards were identified, and the last plant in Zvolen had 49 hazards.

To identify hazards and risks, our team utilised a custom-developed checklist, which enabled us to identify 297 hazards and risks across all operational sites of the enterprise within the working environment. Subsequently, we proceeded with risk analysis and assessment. The risk assessment was conducted using a point-based method and an extended point-based method, employing a five-level scale for probability and consequence, with 5 as the highest value. As a result, multiple hazards, risks, and sources of risk were identified. The complete results for all operational sites are presented in Table 1.

**Figure 3:**
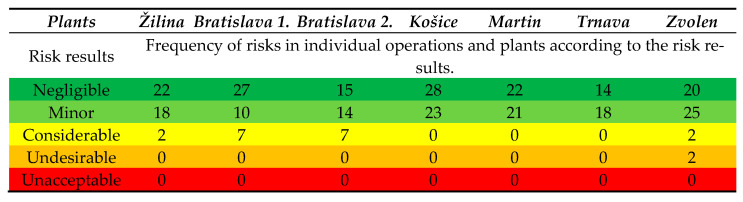
Evaluated risks based on risk outcome for the job position of machine operator (Authors). Colours categorize risk classifications, which are more or less universal for risk assessment in OSH across experts.

The collected data were converted into a risk catalogue, which served as the basis for risk assessment. In this catalogue, risk parameters such as probability and consequence were assigned, providing the necessary quantitative and qualitative data on risk levels (risk magnitude). Additionally, the risk catalogue included proposed measures that each operational site was required to implement within a specified timeframe. In the final step, after implementing these measures, we conducted a reassessment of risks, where probability and consequence parameters were re-evaluated based on the updated workplace conditions and specific risks after mitigation measures had been applied. The evaluated risks based on the risk outcome are presented in Figure 3. This figure illustrates the risk levels before and after the implementation of mitigation measures for each identified hazard.

The risk outcome analysis indicates that no risks were classified as undesirable or unacceptable. However, in terms of risk acceptability classification, some risks were identified as critical (termed “considerable” in the risk outcome assessment). These considerable risks were observed in the following plants and operational sites: Bratislava—Plant 1 and Plant 2, each with seven considerable risks identified; Žilina and Zvolen plants, each with two considerable risks identified. All other risks were categorised as negligible or minor, based on the risk outcome evaluation. The verification process also resulted in a list of proposed measures, which significantly supported the individual operational sites in complying with legal OHS requirements. This, in turn, helped reduce the likelihood and impact of workplace accidents, aligning with the primary objective of the study. The enterprise evaluated the application of the ALrisk model as highly beneficial. One of the most impactful aspects was the supporting component of the ALrisk model, which included joint meetings, communication, and consultations. Through active communication among multiple employees, a more objective risk assessment was achieved, preventing biased or one-sided evaluations of identified risks.

#### Unacceptable Risks in Individual Categories Classified According to the Risk Score for the Job Position of Machine Operator

**Figure 4:**
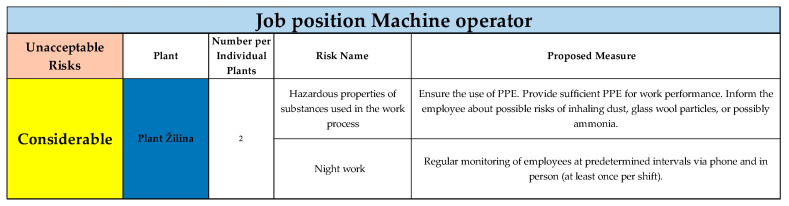
Unacceptable risks in considerable category, Plant Žilina, for job position of machine operator (Authors). Colours in the individual tables are essential for distinguishing the type of plant and the specific category of the resulting risk.

For the job position of machine operator, the risks were evaluated as acceptable in the categories negligible and minor. The classification of unacceptable risks for this job position is presented in Figure 4. Twenty risks were evaluated according to the risk score as unacceptable, classified in three categories—considerable, undesirable, unacceptable.

Within the “considerable” category, 18 risks were assessed. In the category “undesirable”, two risks were assessed. In the final category, “unacceptable”, no hazards or risks were identified and evaluated. For the Žilina plant, two risks were evaluated in the category of considerable risks, for which appropriate measures were proposed.

**Figure 5:**
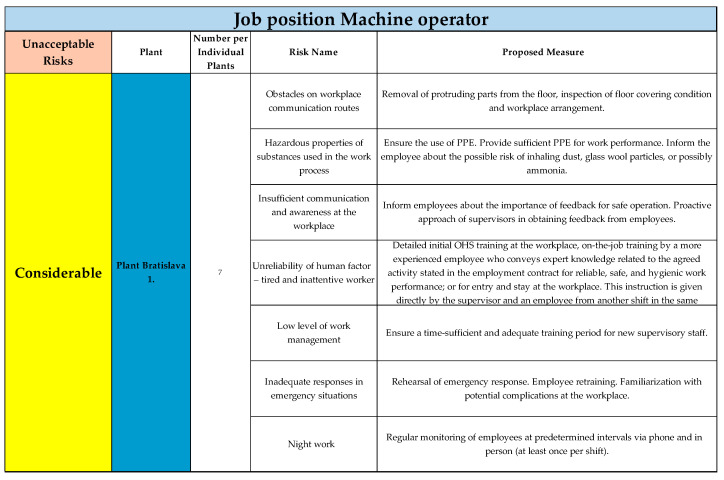
Unacceptable risks in considerable category, Plant Bratislava 1., for job position of Machine operator (Authors). Colours in the individual tables are essential for distinguishing the type of plant and the specific category of the resulting risk.

The results for the Bratislava 1 plant, including seven unacceptable risks in the considerable category, are shown in Figure 5.

**Figure 6:**
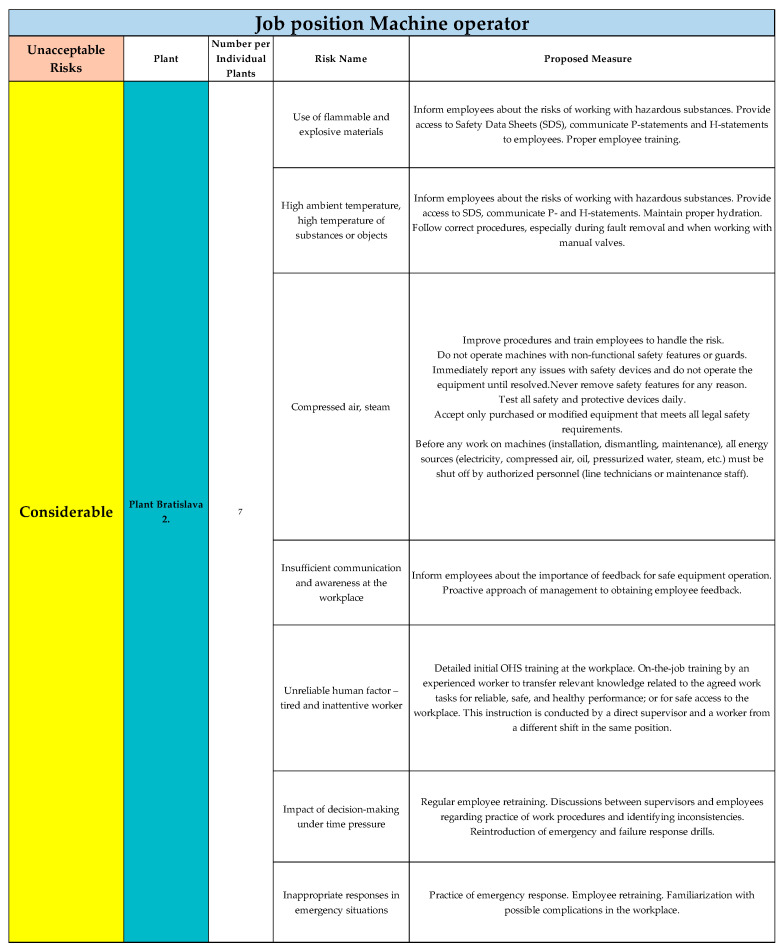
Unacceptable risks in considerable category, Plant Bratislava 2., for job position of Machine operator (Authors). Colours in the individual tables are essential for distinguishing the type of operation and the specific category of the resulting risk.

For Plant Bratislava 2, seven risks were evaluated in the category of considerable risks, for which appropriate measures were proposed. The results for the Bratislava 2 are shown in Figure 6.

**Figure 7:**
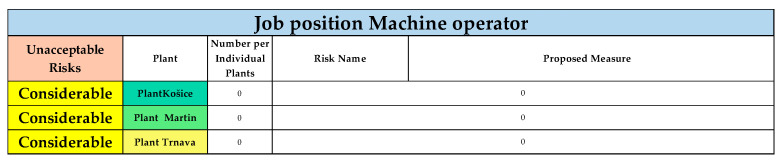
Unacceptable risks in considerable category, Plant Košice, Martin and Trnava, for job position of Machine operator (Authors). Colours in the individual tables are essential for distinguishing the type of operation and the specific category of the resulting risk.

The results for the other plants (Košice, Martin, Trnava) are shown in Figure 7.

For the plants in Košice, Martin, and Trnava, no risks were evaluated in the category of considerable risks.

**Figure 8:**
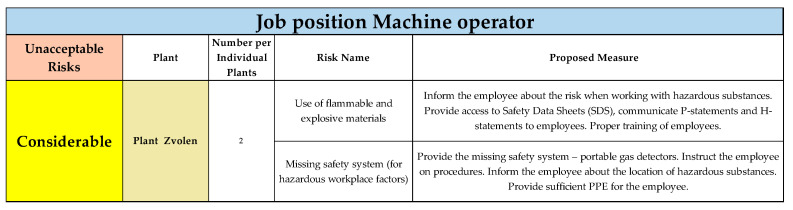
Unacceptable risks in considerable category, Plant Zvolen, for job position of Machine operator (Authors). Colours in the individual tables are essential for distinguishing the type of operation and the specific category of the resulting risk.

The results for the considerable risk category for Plant Zvolen are shown in Figure 8. As the final plant in the category of considerable risks, two risks were evaluated, for which appropriate measures were proposed.

**Figure 9:**
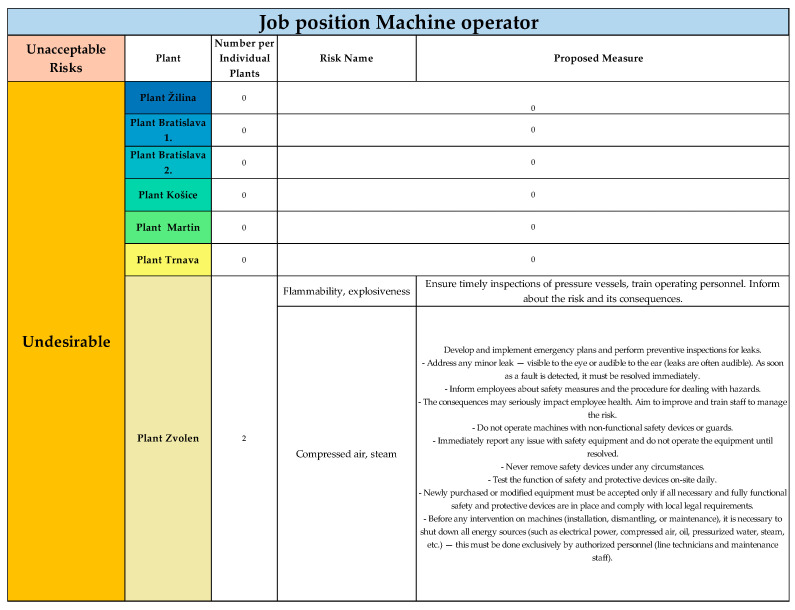
Unacceptable risks in undesirable cathegory, in all plants, for job position of Machine operator (Authors). Colours in the individual tables are essential for distinguishing the type of operation and the specific category of the resulting risk.

The results concerning considerable risks are presented in Figure 9.

Undesirable risks were evaluated according to the risk score in the Zvolen plant, with a total of two risks. Appropriate measures were proposed for these unacceptable risks in the Trnava plant.

**Figure 10:**
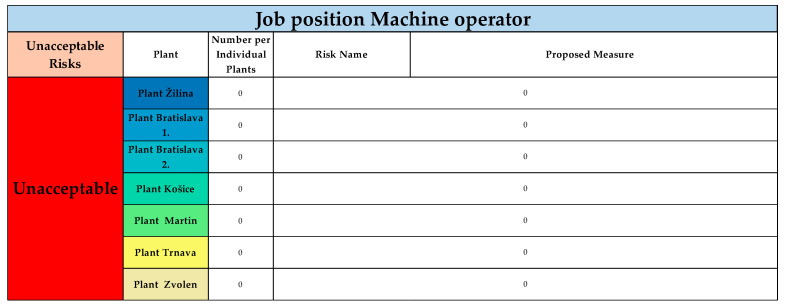
Unacceptable risks in unacceptable cathegory, for all plants, for job position of Machine operator (Authors). Colours in the individual tables are essential for distinguishing the type of operation and the specific category of the resulting risk.

Figure 10 illustrates the results of unacceptable risks for the job position Machine operator.

In the risk score category “unacceptable risks”, no risks were identified or evaluated during the application across all plants for the job position of machine operator.

### 3.3. Evaluation of Key Results from the Verification of the Proposed Model for the Job Position Operator of Heat Exchanger and Homogenisation in a Manufacturing Enterprise

**Figure 11:**
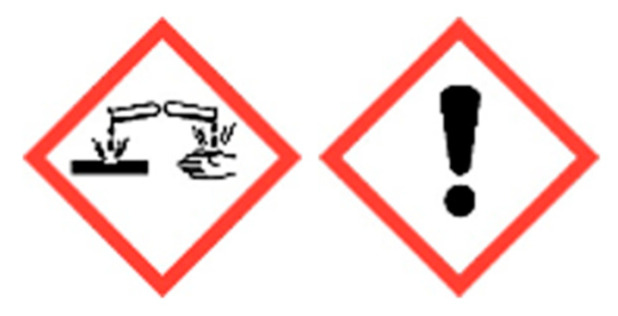
Hazard pictograms of dangerous substances present in the workplace [45].

The job position Operator of a heat exchanger and homogenisation is highly specialised and carries a range of occupational risks affecting employees. This role requires not only technical education and skills but also a qualification course for operating Group A gas equipment. The enterprise where this role is performed manufactures cement and focuses on optimising production processes to enhance efficiency and sustainability. Due to its advanced technology, the company is among the most modern cement plants. It produces cement from domestic raw materials for all types of construction projects, with the entire production process—from quarry blasting to the final product—meeting the highest quality standards. The key responsibilities of this position include operating and monitoring the mechanical and technical equipment of the heat exchanger, overseeing raw meal transportation, homogenisation, electrostatic precipitators, and dust transport, breaking up oversized materials in the kiln (or cooler) using a water cannon, and relaying operational information to the incoming shift worker. The technical skills required include physical cleaning of the calcination channel and base section of the rotary kiln, waste handling and disposal, and the operation of gas equipment. Due to workplace conditions and hazards, the operator is provided with personal protective equipment (PPE), essential for safe work, including helmets, company overalls, a company jacket, gloves, work boots, thermal underwear, thermal socks, a scarf, a vest, a sweatshirt, and a winter coat. Operators work outdoors in all weather conditions and are exposed to both extreme cold and intense heat near the kiln. They handle motorised equipment, work at heights, perform tasks on platforms, adopt awkward postures, and operate in confined spaces with limited movement. Additionally, they work with hazardous substances that can be inhaled, cause burns, or irritate the skin, respiratory tract, and eyes. The work environment is characterised by high noise levels exceeding legal limits. Employees also handle extremely hot materials, which pose a risk of burns, heat exhaustion, or loss of consciousness due to overheating. Other risks include vibrations affecting the upper limbs for more than two-thirds of the working shift, manual handling of heavy loads, and the operation of designated technical equipment. Due to these conditions, the job is classified under the third category of work risk levels. For the Operator of heat exchanger and homogenisation, a total of 68 hazards and risks were identified. The most critical include poor ergonomic factors, exposure to hot substances, inhalation and skin absorption of hazardous materials, restricted movement in confined spaces, and improper or inadequate use of PPE. The most high-risk substances found in the workplace include Portland cement clinker and dust from cement clinker production. These materials carry hazard symbols indicating corrosive, caustic, and irritant properties. According to the Safety Data Sheet (SDS) and H-statements, these substances can cause severe eye damage, irritate the skin, and trigger allergic skin reactions and respiratory irritation. The pictograms of hazardous substances identified in the workplace are shown in Figure 11.

**Figure 12:**
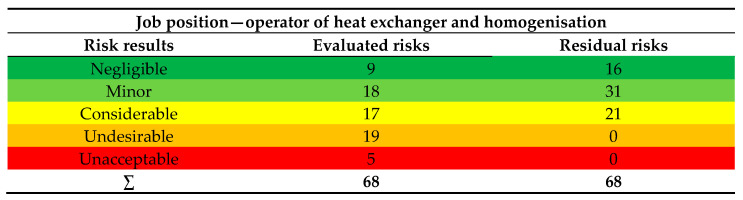
Evaluated risks based on risk outcome for the job position Operator of heat exchanger and homogenisation (Authors). Colours categorize risk classifications, which are more or less universal for risk assessment in OSH across experts.

By assigning numerical values to probability and consequences, we determined the risk levels. The risks were classified as negligible, minor, considerable, undesirable, and unacceptable. Some risks were also identified as non-acceptable. In Phase 1.2 of the ALrisk model—Implementation of the OHS Risk Assessment Process, specifically Step 1.2.5—Reassessment of Risks, it became evident that after defining and implementing mitigation measures, the risks were reduced to an acceptable level. For the job position of operator of heat exchanger and homogenisation, a total of 68 risks were identified and evaluated. The specific numbers of evaluated risks are shown in Figure 12.

For the job position of operator of heat exchanger and homogenisation, a total of 68 risks were identified and evaluated. The specific numbers of evaluated risks are shown in the given image.

#### Unacceptable Risks in Individual Categories Classified According to the Risk Score for the Job Position Operator of Heat Exchanger and Homogenisation

**Figure 13:**
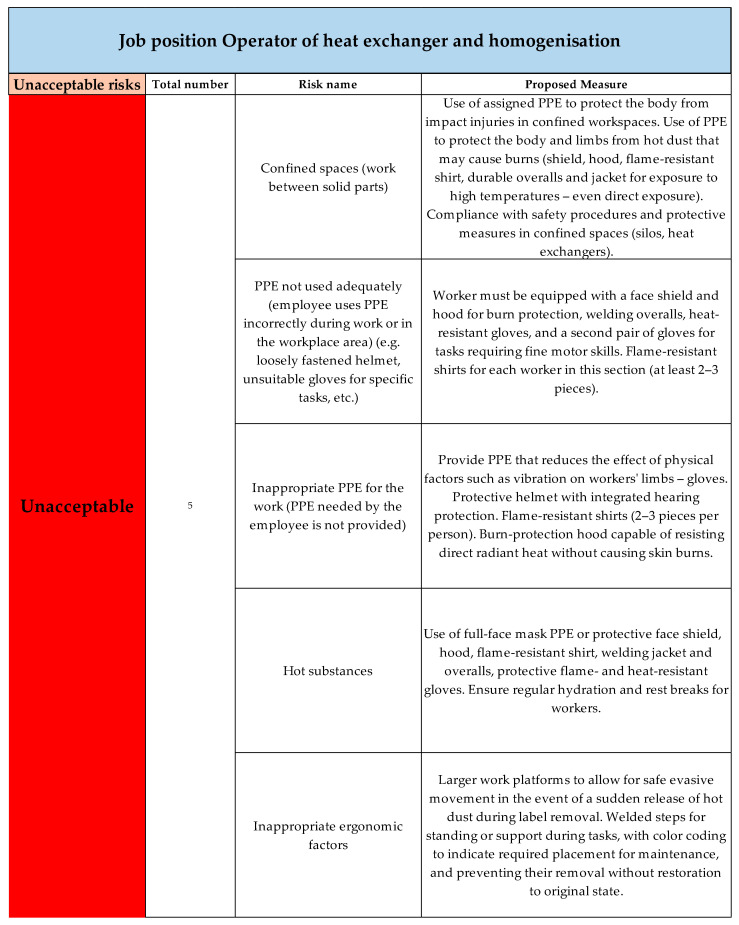
Unacceptable risks for job position Operator of Heat exchanger and homogenization (Authors). Colors in the individual tables are essential for distinguishing the type of operation and the specific category of the resulting risk.

For the job position Operator of heat exchanger and homogenisation, the risks were evaluated as acceptable in the categories negligible and minor. Forty-one risks were evaluated according to the risk score as unacceptable, classified in three categories—considerable, undesirable, unacceptable. The unacceptable risks for job position Operator of heat exchanger and homogenisation are shown in Figure 13.

The image shows the risks evaluated according to the risk score as unacceptable, with a total of five risks. Measures were proposed for these risks to reduce them.

The implementation of the risk assessment process as part of the ALrisk model has significantly contributed to improving safety conditions within the enterprise. This process enabled the company to effectively identify hazards using a customised checklist, specifically designed and adapted for this particular job position. By applying the model, the company successfully minimised potential hazards, ensuring a higher level of employee protection and reducing workplace accidents and occupational diseases. The final outcome, in line with the set objectives, was the improvement of the working environment, an increase in safety and awareness for the Operator of heat exchanger and homogenisation, and a reduction of identified risks to an acceptable level. Furthermore, the process provided new insights that not only positively impacted this specific job position but also benefited the enterprise as a whole. The verification of the process contributed to the following outcomes: enhanced prevention of workplace accidents and occupational diseases, increased efficiency of work processes, greater interest and awareness in workplace safety, emphasis on the necessity of continuous and open communication with employee, and strengthening of overall OHS management, Ultimately, these improvements led to a better safety culture within the organization.

## 4. Discussion

The ALrisk model is designed as an effective and innovative approach that can be seamlessly implemented within an enterprise. It introduces a systematic procedure outlining how to conduct and which steps to follow in the assessment and management of occupational risks. The methods used to identify hazards and threats, as well as the methods applied for risk evaluation, must be determined by the employer or the responsible OHS personnel. The originality of the ALrisk model lies in its innovative and systematic approach to occupational risk management, which is designed to be practical, efficient, and adaptable to various working conditions. Unlike other methodologies, the proposed model provides a structured framework and recommends specific risk assessment methods, allowing companies to select the most suitable tools based on their needs and specific conditions. What truly sets ALrisk apart is its distinctive originality, which differentiates it from other approaches:

1. Flexibility—The ALrisk model can be applied across various industries, company sizes, and specific working conditions, making it highly adaptable.
2. Systematic Approach—It provides clearly defined steps and a structured framework for risk assessment and management, eliminating ambiguity and disorganisation.
3. Practicality—Designed to address the real needs of enterprises, it is easily applicable in practice due to its detailed structure, including its supporting components, main sections, phases, and steps.
4. Innovation—The model is research-based and integrates modern technologies, methodologies, and online tools for risk assessment, such as the OiRA web platform.
5. Dynamism—ALrisk is not a static document but a living process that can be continuously updated, improved, and adapted to emerging risks.
6. Predictive Capability—Beyond assessing current risks, the model’s supporting and main components allow companies to anticipate future hazards, threats, and risks and implement preventive measures accordingly.
7. Proactive Approach—The model does not merely address problems but focuses on preventing them and strategic safety planning.
8. Efficiency—ALrisk reduces redundancy in risk assessment and management, saving both time and costs for enterprises.
9. Adaptability—The model functions effectively under various working conditions and is designed to respond to legal and technical changes.

Enterprises that choose to implement the ALrisk model gain a practical, systematic, and adaptable tool for occupational risk assessment and management. This enables them not only to ensure employee safety but also to minimise costs associated with workplace accidents and maintain compliance with legal requirements. The ALrisk model is not just another risk assessment method; it is a comprehensive solution that helps businesses approach workplace safety strategically, systematically, and in full compliance with regulations.

The ALrisk model provides a systematic and flexible framework for occupational risk assessment, adaptable to various working conditions. Unlike methods primarily focused on risk quantification, such as the cost estimation method for OHS [24] or the point-based assessment of probability and consequences [26], ALrisk emphasises comprehensive risk identification and management. Compared to approaches using specific assessment tools, such as the Fine–Kinney method [27] or fuzzy logic [31], ALrisk offers a predictive approach, allowing employers to adjust the risk assessment process according to their specific needs. A key distinction is its integration with ISO 31000:2018 and ISO 45001:2018 standards, ensuring compatibility with internationally recognised risk management principles [34,35]. While models like the Workplace Risk Assessment Workbook [9] rely on fixed checklists, ALrisk provides greater flexibility in selecting assessment methods, which is particularly beneficial for enterprises operating in dynamic work environments. Unlike traditional methods such as the bow-tie approach [30], or the Analytic Hierarchy Process (AHP) [8], ALrisk differentiates itself by focusing on supporting processes, including active communication with employees, continuous monitoring, and consultations during the implementation of safety measures. This comprehensive approach not only enables effective risk identification and assessment but also facilitates better planning and implementation of measures to eliminate risks.

## 5. Conclusions

The article presents a model for the assessment and management of risks in large and medium-sized enterprises, which was verified through two applications. The main challenges in their implementation were the correct setting of the application of individual steps and explaining them to the companies and employees where it was carried out. Their involvement is the basis for the success of the analysis performed. A challenge was also the alignment of responses in expert evaluations from job position representatives and safety technicians. Their views on the use of PPE identified through the analysis differed.

When creating a unified model for several plants within the holding—seven companies—the level and degree of safety culture varied. This issue could be resolved through regular meetings of safety technicians operating in different plants and through discussion and the search for common solutions.

The article focuses on the application of the first part of the ALrisk model, specifically on the systematic assessment of occupational risks for two specific job positions within manufacturing enterprises. This phase of the model includes steps from risk identification to the proposal of measures and the re-evaluation of risks. Due to the scope of the article and its targeted focus on this part of the model, the second part of the model aimed at risk management—which includes employee training and monitoring the effectiveness of implemented changes—is not the subject of this publication.

The ALrisk model has been applied to multiple job positions, demonstrating its originality and flexibility. One of the key findings of this article is the verification of the ALrisk model for two specific job roles: operator of heat exchanger and homogenisation. These two positions are fundamentally different and operate under completely distinct working conditions. The results were obtained through the application of the ALrisk model for both roles in different industries and company sizes. The research was conducted during the development of the ALrisk model, aiming to enhance the efficiency and innovation of occupational health and safety (OHS) management within enterprises.

It can be stated that the job position of machine operator was present in seven operational sites across six plants within the assessed enterprise. The machine operator role is exposed to multiple occupational risks, with the most considerable identified hazards including flammability and explosiveness, hazardous properties of substances used in the work process, and compressed air and steam. Other critical hazards were classified as obstacles in workplace pathways, high environmental or material temperatures, lack of safety systems, inadequate communication, human factor unreliability, decision-making under time pressure, low level of work management, improper reactions in emergency situations, and night shifts. The risks were subsequently evaluated based on their severity and outcome classification, with undesirable and considerable risks being deemed unacceptable. During the identification, analysis, and assessment process, additional risks were classified as acceptable, including negligible and minor risks. No risks within this job position were categorized as unacceptable on the highest risk scale. Before the actual application of the ALrisk model to the job position of machine operator, there was a request to unify the risk assessment process across all operations of the company, as previously non-unified and locally adjusted methodologies were used. The results after applying the model were as follows:

1. The ALrisk model made it possible to unify risk assessment procedures in seven operations and six plants in Slovakia,
2. Standardisation of the job title terminology was achieved, which had previously been recorded in various forms (e.g., machine operator–technician, machine operator–mechanic, machine operator–walker), and were merged under the unified designation “machine operator”,
3. Employees’ trust in the OHS system increased, as they perceived that a consistent and thorough approach to the protection of their health and safety was being applied.

The second specific job position, Operator of heat exchanger and homogenisation, was assessed in a single enterprise. The primary findings from the risk assessment process included the following considerable identified hazards: confined spaces in the workplace, exposure to hot substances, inappropriate ergonomic factors, and improper or inadequate use of PPE. Other major hazards included inhalation, exposure, or skin absorption of hazardous substances, the presence of airborne material particles, and noise and vibrations. These hazards were identified through a customised checklist specifically designed for this job position and were subsequently analysed. Using the point-based method, risks were assessed and assigned risk levels based on qualitative and quantitative risk outcome evaluation. The first risk assessment results identified 5 unacceptable risks, 19 undesirable risks, and 17 considerable risks. The application of the ALrisk model for the job position operator of heat exchanger and homogenisation, which belonged to the most high-risk positions in the entire company, brought specific results. The aim of the model’s implementation was to improve occupational safety, uncover hidden deficiencies, and introduce a sustainable, systematic risk assessment procedure that would directly support employee health protection and accident reduction. After applying the ALrisk model, the following results were achieved:

1. The job position was reclassified from category 2 to category 3 based on the identified exceeded limits (e.g., noise, vibrations),
2. The model ensured a clear step-by-step process, which enabled managers to proceed systematically without omitting any steps,
3. Specific risks were identified and adequate measures were proposed for their elimination,
4. A safety manual was proposed for the job position, serving as a clear document outlining the most serious risks in the workplace, necessary personal protective equipment, and other information about the job position,
5. An information safety brochure was created for the company, intended for visitors to provide them with basic information about OHS rules within the company.

With this, it can be confirmed that the application itself brought many benefits to both companies. The application ensured comprehensive compliance with correct safety procedures and legal regulations for companies in the area of OHS. The feedback after implementation and presentation of the results to the companies was significantly positive. The results were positively evaluated not only by managers, but also by employees working in the respective job positions.

The processed results are significant for occupational health and safety (OHS) managers, senior employees, as well as employers and professionals in the field of OHS, with a focus on risk assessment and workplace risk management. The research is also beneficial for specialists seeking information on workplace risk assessment and management, as well as the effective integration of modern technologies and online tools for risk evaluation. The findings can serve as a key basis for developing documentation and internal company guidelines for OHS management in enterprises. The results are also valuable for employee training or for industry professionals who are keen on continuous learning and adopting innovative and systematic approaches as an outcome of research and development in OHS management. From a theoretical perspective, however, the article contributes to the methodological framework of risk assessment by offering an innovative, structured, and practically verified approach that is applicable across various types of operations. At the same time, the results reflect practical barriers in larger enterprises, while the recommendations resulting from the application of the model are potentially transferable to the environment of smaller enterprises.

The findings obtained from qualitative and quantitative research possess specific characteristics. The research was conducted across large, medium-sized, and small enterprises. The ALrisk model was developed based on an analysis of the current state of the studied issue, existing risk assessment procedures in the EU and Slovakia, contemporary methodologies, insights from experts and theorists in the field of occupational health and safety (OHS), practical knowledge and skills from enterprises, data from the European ESENER surveys (1, 2, 3), information from the National Labour Inspectorate, evaluated statistical data, and other relevant sources. During the OHS risk assessment for the position of machine operator, six team members participated in the evaluation. For the risk assessment of Operator of heat exchanger and homogenisation position, eight team members were involved. Over time, the risk assessment for the machine operator position was conducted across all operations for a period of two years, while the risk assessment for the Operator of heat exchanger and homogenisation position lasted five months.

The next focus of research activities will be the verification of the second main component of the proposed ALrisk model, which is oriented towards OHS risk management. The area of employee training as part of comprehensive risk management will be the subject of further research following this article. An essential aspect will be the expansion of the model’s verification to additional manufacturing enterprises and sectors, ensuring a broader database of data to enhance the process. Future research will concentrate on addressing current practical challenges that enterprises most frequently face in relation to workplace risk assessment and management. A key source of information will be the new European ESENER 4 survey, which will provide up-to-date data for research in the field of OHS.

## Figures and Tables

**Table 1:** Hazards, threats, and sources of risk in the workplace.

Hazards, Threats, and Sources of Risk in the Workplace
***Job position: Machine operator***
Machine and vehicle movement
Flammability, explosiveness
Improper workplace layout
Sharp edges, corners, points, rough surfaces, protruding parts Working at heights Obstacles in workplace pathways Electrical switches of machines Impact of portable electrical devices Dangerous properties of substances used in the work process Use of flammable and explosive materials High temperature of the environment, high temperature of materials or objects Compressed air, steam Presence of allergens Insufficient communication and awareness in the workplace Unreliable human factor—tired and distracted workers Impact of decision-making under time pressure Night work Workplace rotation Manual handling of loads Pressure equipment Gas equipment

## Data Availability

The data presented in this study are available on request from the corresponding author due to (privacy, security reasons).

## Abbreviations

The following abbreviations are used in this manuscript:

AHP	Analytic Hierarchy Process
DTE	Designated Technical Equipment
ETA	Event Tree Analysis
ESENER	European Survey of Enterprises on New and Emerging Risks
EU-OSHA	European Agency for Safety and Health at Work
FMEA	Failure Mode and Effects Analysis
FTA	Fault Tree Analysis
HAZOP	Hazard and Operability Study
ISO	International Organization for Standardization
MSDs	Musculoskeletal Disorders
OHS	Occupational Health and Safety
OiRA	Online Interactive Risk Assessment
PPE	Personal Protective Equipment
SDS	Safety Data Sheet
SMEs	Small and Medium-Sized Enterprises
